# Effects of regular physical exercise on ejaculatory control: a comparative and interventional study among male university students

**DOI:** 10.3389/fpubh.2026.1820347

**Published:** 2026-06-11

**Authors:** Jiedong Zhou, Min Liu, Shian Hu, Yong Ouyang, Yucheng Kong

**Affiliations:** 1 First Affiliated Hospital of Gannan Medical University, Ganzhou, China; 2 Gannan Medical University, Ganzhou, China

**Keywords:** college students, IELT, physical exercise, premature ejaculation, sexual function

## Abstract

**Background:**

Intravaginal ejaculatory latency time (IELT) is a key indicator for evaluating premature ejaculation (PE). While physical exercise is beneficial for male sexual health, evidence specifically linking structured exercise programs to IELT improvement is limited.

**Aim:**

To compare IELT between athletes and non-athletes, and to examine whether a 3-month guided structured exercise program improves IELT in non-athletes.

**Methods:**

A mixed-design study included 67 male university students (30 athletes, 37 non-athletes). Non-athletes underwent a 3-month, guided, home-based structured exercise program. IELT was assessed via self-report.

**Outcomes:**

Primary outcome was change in IELT; secondary outcomes included PE prevalence and regression modeling.

**Results:**

Athletes had significantly longer median IELT than non-athletes (8.0 vs. 5.0 min, *P* = 0.003). Among adherent non-athletes (*n* = 20), median IELT increased from 5.0 to 6.25 min post-intervention (*P* < 0.001). Exercise frequency was the strongest predictor of IELT (β = 0.41, *P* = 0.002).

**Clinical implications:**

Guided structured exercise program may serve as a feasible, non-pharmacological candidate strategy for improving ejaculatory control in young men, though its efficacy requires confirmation in randomized controlled trials.

**Conclusion:**

Regular physical activity is associated with longer IELT. Preliminary findings suggest that a brief guided structured exercise program is associated with IELT improvement in non-athletes, though causal inference is limited by the lack of a concurrent control group. However, these preliminary findings require confirmation in larger, adequately powered randomized controlled trials due to the limited sample size and high attrition rate in this exploratory study.

## 1 Introduction

Intravaginal ejaculatory latency time (IELT) is currently recognized as the most objective and standardized measure for diagnosing and assessing premature ejaculation (PE) (1). According to the consensus of international sexual medicine societies, lifelong and acquired PE are primarily distinguished by the timing of symptom onset (lifelong vs. acquired), with IELT serving as a key supportive criterion. Commonly referenced clinical thresholds include an IELT of approximately 1 min for lifelong PE and 3 min or less for acquired PE (2). As a key objective indicator of male sexual function, IELT is influenced by multiple interacting factors, including physiological, psychological, behavioral, and lifestyle components. Recent bibliometric analysis of the PE literature over the past two decades confirms that research interest in ejaculatory dysfunction has grown substantially, with evolving hotspots ranging from pharmacological treatment to behavioral and lifestyle interventions (3).Therefore, a comprehensive understanding and effective management of male sexual health require an integrated approach that considers all these dimensions (4, 5).

In recent years, increasing attention has been focused on the role of physical exercise in male sexual health. Both aerobic exercise (such as running and swimming) and resistance training have been shown to significantly improve erectile function and sexual satisfaction (6). Regular exercise can enhance sexual function through multiple mechanisms, including improved cardiovascular capacity, enhanced blood circulation, regulation of endocrine hormones, and reduction of anxiety and depression (7, 8). Furthermore, physical training may improve overall fitness and boost sexual confidence, thereby indirectly prolonging IELT and serving as an important adjunctive strategy for managing sexual dysfunction (9).

Although existing evidence supports the beneficial effects of exercise on male sexual function, comparative studies examining differences in IELT among populations with varying exercise habits remain limited. College athletes, who engage in frequent physical activity, typically exhibit superior physical fitness, lower stress levels, and better overall sexual health. In contrast, non-athletes, who often lead sedentary lifestyles and experience higher psychological stress, are more likely to have shorter IELT and related dysfunctions (10, 11). Regular exercise may enhance sexual function by increasing testosterone levels and optimizing endocrine balance, whereas physical inactivity can cause hormonal fluctuations and sexual dysfunction (12).

Based on these considerations, the present study aimed to compare IELT between college athletes and non-athletes and to further evaluate the effects of regular exercise interventions on IELT among non-athletes. We hypothesized that athletes would exhibit significantly longer IELT than non-athletes and that a structured exercise program could effectively prolong IELT in non-athletes. The findings of this study are expected to provide empirical evidence for the role of physical exercise in regulating male sexual function and to offer a scientific basis for promoting sexual health education and interventions among college students.

## 2 Materials and methods

### 2.1 Study design

This study employed a mixed-design, combining a cross-sectional comparison with a prospective intervention. The first phase involved a cross-sectional survey comparing IELT between college athletes and non-athletes. The second phase implemented a 3-month guided structured exercise program intervention among non-athletes to evaluate the effect of the structured exercise program on IELT improvement.

### 2.2 Participants

Participants were recruited between May 2025 and July 2025. Participants were recruited from universities in southern Jiangxi Province, China, including both physical education institutions and general universities. Recruitment was conducted through public announcements on campus bulletin boards, student websites, online forums, and social media, as well as via email and telephone invitations. Additionally, volunteers were recruited from university health clinics through brief presentations about the study.

All procedures involving human participants were conducted in accordance with the ethical standards of the institutional and/or national research committee and with the 1964 Helsinki Declaration and its later amendments or comparable ethical standards. Written informed consent was obtained from all individual participants included in the study.

Inclusion Criteria: Eligible participants were male college students aged 18–28 years who met the following criteria: (1) maintained a steady heterosexual relationship, defined as cohabitation or engaging in regular sexual intercourse at least once per week, to ensure reliable IELT data collection; (2) reported good health without any major physical illnesses; and (3) voluntarily agreed to participate and provided written informed consent.

**Figure 1:**
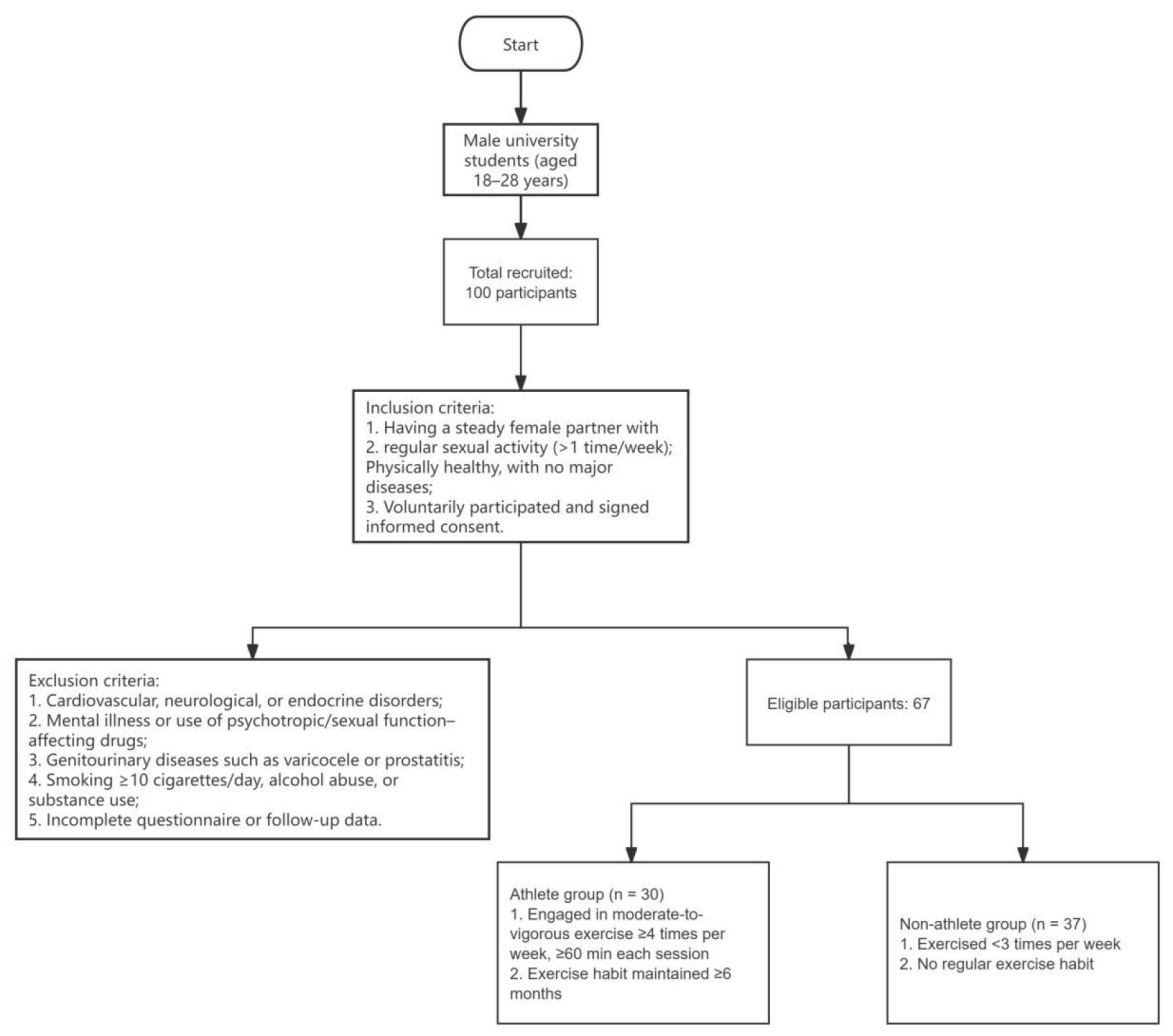
Flowchart of participant selection. A total of 100 male college students were initially recruited. After applying the inclusion and exclusion criteria, 67 eligible participants were included in the final analysis, comprising 30 athletes and 37 non-athletes.

Exclusion Criteria: Participants were excluded if they met any of the following criteria: (1) presence of severe chronic diseases affecting sexual function, such as cardiovascular, neurological, or endocrine disorders (e.g., diabetes mellitus, thyroid dysfunction); (2) history of psychiatric disorders (e.g., major depression or anxiety disorders) or current use of psychotropic medications; (3) known urogenital conditions affecting sexual function (e.g., prostatitis, varicocele, penile anatomical abnormalities); (4) use of medications that could influence sexual function within the past 3 months, including but not limited to antidepressants (e.g., SSRIs), dapoxetine, or topical anesthetics specifically used for delaying ejaculation. Use of any such medications during the study period was not allowed; (5) history of heavy smoking (≥10 cigarettes per day), alcohol dependence, or substance abuse; (6) inability to complete questionnaires or follow study procedures, resulting in incomplete data. The participant selection process is shown in Figure 1.

### 2.3 Ethical approval and consent

This study was conducted in accordance with the Declaration of Helsinki and was reviewed and approved by the Ethics Committee of the First Affiliated Hospital of Gannan Medical University (Approval No.LLSC-2025-164). The study was prospectively registered with the Thai Clinical Trials Registry (TCTR20250513005). TCTR was selected as it is a WHO Primary Registry providing an efficient English-language registration process that fully complies with ICMJE requirements, which is suitable for international publication. Written informed consent was obtained from all individual participants included in the study after a full explanation of its purpose and procedures. Confidentiality was strictly maintained, and participants could withdraw at any time without penalty.

Group Definition: In addition to the general criteria, specific definitions were applied to differentiate between the two study groups.

Athletes (Sports Students): Participants enrolled in a physical education major who met the criteria for regular exercise, defined as engaging in moderate-to-vigorous physical activity at least four times per week for 60 min or more per session, consistently for at least 6 months.

Non-athletes are defined: As participants who engage in regular physical exercise fewer than three times per week. For the intervention phase, non-athletes were also required to be willing and able to participate in a 3-month structured exercise program following the prescribed regimen.

### 2.4 Data collection

Demographic and general information were collected using a structured questionnaire. The variables gathered included age, height, weight, body mass index (BMI), lifestyle factors (such as smoking history and late-night habits), frequency of sexual intercourse per week, duration of cohabitation or relationship, psychological status, and household registration type (urban or rural). Psychological Stress: Assessed using the 10-item Perceived Stress Scale (PSS-10), with higher scores indicating greater stress. Sexual intercourse frequency (times/week) was also assessed at baseline and monthly during the intervention period to control for its potential independent effect on IELT.

Sleep Habit: Assessed by self-reported average nightly sleep duration and a single-item question on sleep quality (1 = very poor to 5 = very good). Physical Activity Level (for athletes): Verified by training records provided by their coaches.

IELT was assessed using a retrospective self-reported questionnaire. We acknowledge that retrospective self-report is susceptible to recall bias and is less precise than prospective stopwatch measurements, though it remains the most practical and widely used method in current sexual medicine research. Participants were asked to anonymously report their average IELT—defined as the time interval between vaginal penetration and ejaculation—based on their sexual experiences over the past 3 months. To enhance data accuracy, participants were instructed to estimate their average IELT rather than relying on a single sexual episode. According to established clinical standards, an IELT shorter than 1–3 min indicates premature ejaculation, while the mean IELT in the general population is approximately 6 min. For the purpose of prevalence analysis in this study, PE was operationally defined based solely on an IELT threshold of ≤ 3 min. We acknowledge that this definition does not fully encompass the ISSM (International Society for Sexual Medicine) standard criteria, which also require a lack of perceived ejaculatory control and resultant personal distress. Therefore, the PE classification herein primarily refers to a short ejaculatory latency rather than a strict clinical diagnosis. Self-reported IELT, although prone to recall bias, is widely used in sexual medicine research and shows a strong correlation with stopwatch-timed IELT (*r* = 0.70–0.85 in previous validation studies). To minimize recall error, participants were instructed to report mean IELT based on at least three recent intercourse events. Exercise frequency was self-reported for all participants; for athletes, self-report was cross-checked against coach-provided training records when available.

### 2.5 Guided structured exercise program intervention

Non-athletes in the intervention phase undertook a 3-month, guided, home-based structured exercise program designed to increase overall physical activity in a feasible and scalable manner for university students. The program emphasized moderate-to-vigorous aerobic exercise, supplemented by basic resistance training, and was delivered with standardized instructions and remote adherence support.

#### 2.5.1 Initial education and instruction

All participants attended a single 60-min group session led by a trained research team member. The session covered: (1) health benefits and safety principles of regular exercise; (2) how to monitor exercise intensity using the Borg Rating of Perceived Exertion (RPE) scale and/or heart-rate targets; (3) demonstration of the aerobic and resistance exercises included in the program; and (4) how to complete exercise logs and report adherence.

#### 2.5.2 Prescribed exercise regimen (FITT)

Frequency: ≥4 sessions per week (target 5 sessions/week).

Intensity: moderate-to-vigorous intensity, defined as RPE 12–16 (or approximately 64–95% of age-predicted HRmax when heart-rate monitoring was available).

Time: 45–60 min per session.

Type:

Aerobic component (primary): running/jogging, brisk walking, cycling, swimming, or equivalent continuous aerobic activity, performed for 30–45 min per session.

Resistance component (supplementary): 10–15 min of bodyweight or light resistance exercises (e.g., squats, lunges, push-ups, planks), 2–3 sets of 8–12 repetitions for major muscle groups.

Progression: Participants were instructed to begin at the lower end of the prescribed duration and intensity during weeks 1–2 and then progressively increase weekly exercise volume by approximately 5–10% every 2 weeks, primarily by extending aerobic duration and/or increasing intensity within the prescribed range, while maintaining safety and tolerability.

#### 2.5.3 Adherence support and monitoring

Adherence was supported through: (1) a structured exercise diary documenting exercise type, duration, and perceived intensity; (2) weekly brief check-ins via a messaging app to address barriers and encourage completion; and (3) optional submission of screenshots or records from exercise apps/wearables when available.

Adherence Criteria: For per-protocol analysis, participants were required to submit logs for ≥70% of study weeks and to report completing ≥4 exercise sessions per week on average across the 3-month period.

### 2.6 Statistical analysis

Given the exploratory nature of this study and the lack of prior effect size estimates for IELT changes following exercise in this population, a formal sample size calculation was not performed a priori. We aimed to recruit a convenience sample of at least 30 participants per group based on logistical feasibility, which is comparable to previous pilot interventional studies in sexual medicine. All statistical analyses were performed using IBM SPSS Statistics for Windows, Version 26.0 (IBM Corp., Armonk, NY, USA). Continuous variables were expressed as mean ± standard deviation (SD) or median with interquartile range (IQR), depending on their distribution, while categorical variables were presented as frequencies and percentages. Comparisons between athletes and non-athletes were conducted using the independent-samples *t*-test or the Mann–Whitney *U* test for continuous variables, and the chi-square test for categorical variables. Changes in IELT before and after the 3-month exercise intervention among non-athletes were analyzed using the Wilcoxon signed-rank test, as the data were not normally distributed. Multivariate linear regression analysis was conducted to examine the independent associations between IELT and potential influencing factors, including exercise frequency, psychological stress levels, sleep habits, and BMI. Prior to modeling, IELT underwent log-transformation to satisfy the assumption of residual normality. Model diagnostics showed VIF all <2.0, ruling out significant multicollinearity. All statistical tests were two-tailed, and a *p*-value less than 0.05 was considered statistically significant.

## 3 Results

### 3.1 Participant characteristics

A total of 67 participants (30 athletes and 37 non-athletes) were included in the cross-sectional analysis. As shown in Table 1, continuous variables—such as age, height, weight, BMI, sleep habits, frequency of sexual intercourse, duration of cohabitation, and years of university enrollment—were expressed as medians (IQR), while categorical variables, including smoking history and household registration, were presented as frequencies and percentages. Shapiro-Wilk test indicated that IELT data were not normally distributed in either group (*P* < 0.05). Accordingly, non-parametric tests (Mann–Whitney *U*, Wilcoxon signed-rank) were applied throughout.

**Table 1:** Comparison of general characteristics and IELT between athletes and non-athletes.

Variable	Athletes (*n* = 30)	Non-athletes (*n* = 37)	U/χ^2^	*P*-value
Age (years)	20.6 (19.5–21.7)	21.0 (20.0–22.0)	472	0.29
Height (cm)	177.0 (172.0–182.0)	174.0 (169.0–178.0)	631	0.34
Weight (kg)	68.5 (63.8–73.0)	71.8 (67.0–76.8)	456.5	0.22
BMI (kg/m^2^)	21.8 (20.4–23.0)	23.7 (22.4–25.2)	431	0.12
Smoking history (yes, %)	5 (16.7%)	7 (18.9%)	0.06	0.82
Late-night frequency (times/week)	3.0 (2.0–4.0)	3.3 (2.0–4.0)	500	0.55
Sexual intercourse frequency (times/week)	2.4 (1.8–3.0)	2.3 (1.7–2.9)	548	0.412
Duration of cohabitation (months)	9.5 (5.0–14.0)	8.8 (4.0–13.0)	588	0.68
Household registration (urban/rural)	17 (56.7%)	23 (62.2%)	0.04	0.84
Years of university enrollment	1.9 (1.2, 2.6)	2.0 (1.3, 2.7)	13.5	0.44
IELT (minutes)	8.0 (6.0–11.8)	5.0 (3.5–7.0)	270.0	0.003^*^

^*^P < 0.05.

Continuous variables are presented as median (interquartile range); categorical variables as frequency (percentage).

IELT, intravaginal ejaculatory latency time; BMI, body mass index.

For most baseline characteristics, no statistically significant differences were observed between the athlete and non-athlete groups (*P* > 0.05). Notably, the median BMI was lower in athletes (21.8 kg/m^2^) than in non-athletes (23.7 kg/m^2^); although not statistically significant (U = 431, *P* = 0.12), this numerical difference likely reflects expected physiological differences between the groups. The two groups were comparable across most demographic, physiological, and behavioral characteristics (all *P* > 0.05), except for IELT, which was significantly longer in athletes (*P* = 0.003).

### 3.2 Comparison of IELT and premature ejaculation prevalence between athletes and non-athletes

#### 3.2.1 Comparison of IELT between athletes and non-athletes

**Figure 2:**
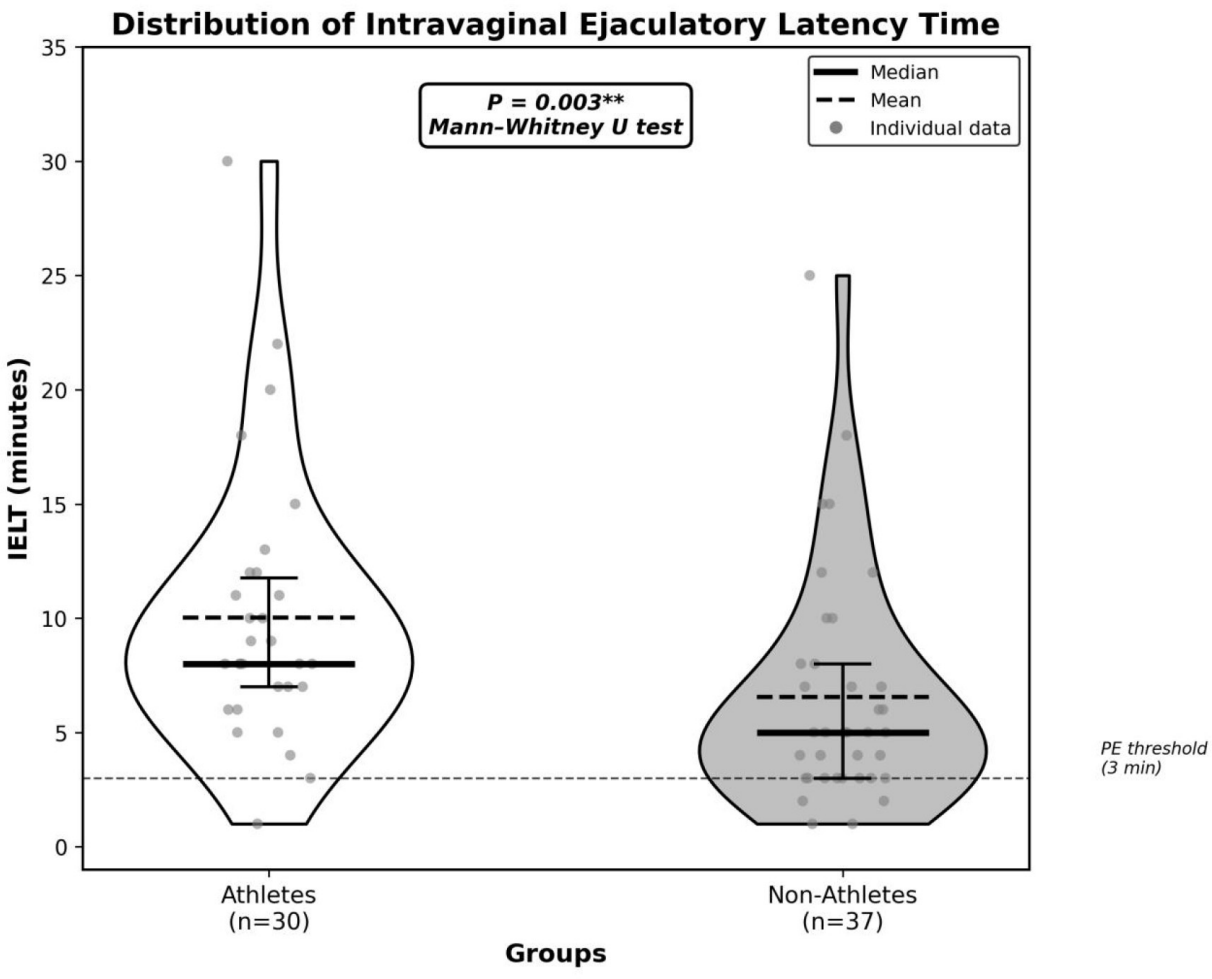
Comparison of IELT between athletes and non-athletes. Data presented as median (IQR). Mann–Whitney *U* = 270.0, *P* = 0.003 (**). PE threshold = 3 min (dashed line).

**Figure 3:**
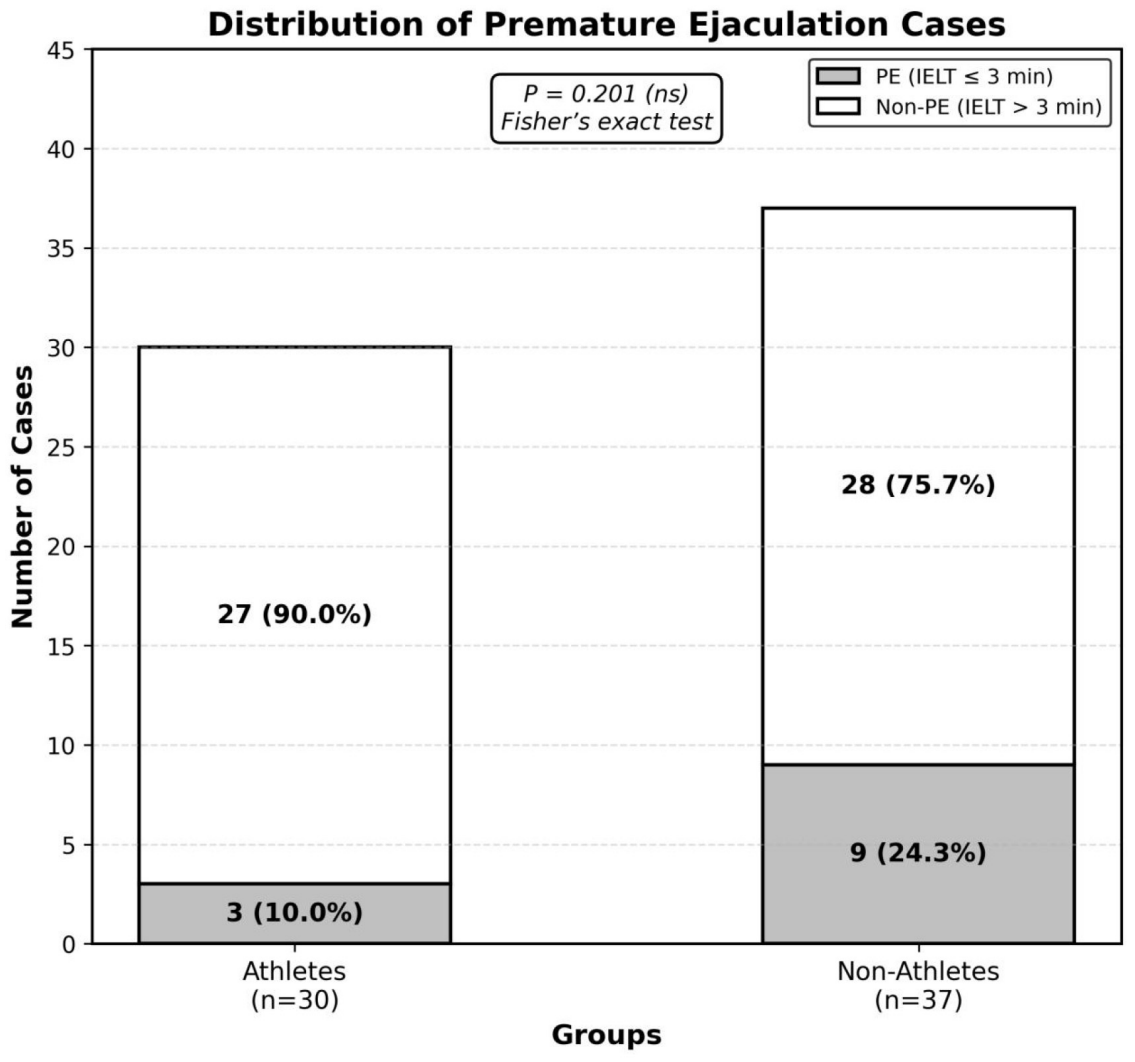
Distribution of premature ejaculation prevalence among athletes and non-athletes. A stacked bar chart illustrates the distribution of premature ejaculation (PE) and non-PE cases in the athlete group (*n* = 30) and the non-athlete group (*n* = 37). PE was defined as an IELT of 3 min or less. Gray bars represent PE cases, while white bars represent non-PE cases. In the athlete group, there were 3 PE cases (10.0%) and 27 non-PE cases (90.0%), compared to 9 PE cases (24.3%) and 28 non-PE cases (75.7%) in the non-athlete group. Although the prevalence of PE was higher among non-athletes, the difference was not statistically significant (*P* = 0.201, Fisher's exact test).

The distribution of IELT among athletes (*n* = 30, blue) and non-athletes (*n* = 37, orange) is illustrated in a violin plot that includes individual data points (Figure 2). The shape of the violin represents the probability density of IELT values. In each plot, the solid red line indicates the median, while the dashed blue line represents the mean IELT. Using the Mann–Whitney *U* test, the median IELT in the athlete group [8.0 min; interquartile range (IQR): 6.25–11.75 min] was significantly longer than that in the non-athlete group (5.0 min; IQR: 3.5–7.5 min; *P* = 0.003). The horizontal dashed line at 3 min denotes the clinical threshold for PE. Abbreviations: IELT, intravaginal ejaculatory latency time; IQR, interquartile range; PE, premature ejaculation (Figure 3). 

#### 3.2.2 Changes in IELT before and after exercise intervention among non-athletes

During the 3-month intervention, median sexual intercourse frequency remained stable among adherent non-athletes [baseline: 2.3 times/week [IQR: 1.7–2.9]; month 3: 2.4 times/week [IQR: 1.8–3.0]], with no significant change (*P* = 0.56). No significant difference in intercourse frequency was observed between athletes and non-athletes at baseline (*P* = 0.41).

Non-athletes who adhered to the guided structured exercise program (meeting adherence criteria, *n* = 20) were included in the per-protocol analysis. It should be noted that the intervention group experienced a relatively high attrition rate (17 out of 37 dropouts, ~46%), which may introduce attrition bias and limit the generalizability of the per-protocol findings. If dropouts derived less benefit, the observed effect size might be overestimated. As detailed in Methods 2.5, the intervention consisted of a home-based structured exercise program prescribed at ≥4 sessions per week, with weekly remote supervision over 3 months.

Before training, the median IELT was 5.0 min (IQR: 3.75–6.0). After 3 months of training, the median IELT increased to 6.25 min. The IQR also widened following the intervention, indicating greater variability in ejaculatory control. Monthly median IELT values progressively increased: 5.5 min at month 1, 5.9 min at month 2, and 6.25 min at month 3. The Wilcoxon signed-rank test confirmed a statistically significant improvement from baseline to month 3 (Z = −3.92, *P* < 0.001), corresponding to a 25% median increase. The 95% confidence interval for the median difference (CI: 15%−35%) was calculated using the Hodges-Lehmann estimator. Monthly IELT values are presented descriptively to illustrate temporal trends; statistical inference was restricted to the baseline-to-month 3 comparison due to the exploratory nature of the analysis.

#### 3.2.3 Subgroup analysis of participants with baseline IELT ≤ 3 min

A *post-hoc* subgroup analysis was conducted on adherent non-athletes whose baseline IELT met the operational PE threshold (IELT ≤ 3 min, *n* = 6). In this small subgroup, the median IELT increased from 2.5 min (IQR: 1.5–3.0) at baseline to 3.5 min (IQR: 2.5–4.5) at month 3. Although numerical improvements were observed, meaningful statistical testing could not be performed due to the extremely small sample size, and these results should be interpreted with caution.

**Figure 4:**
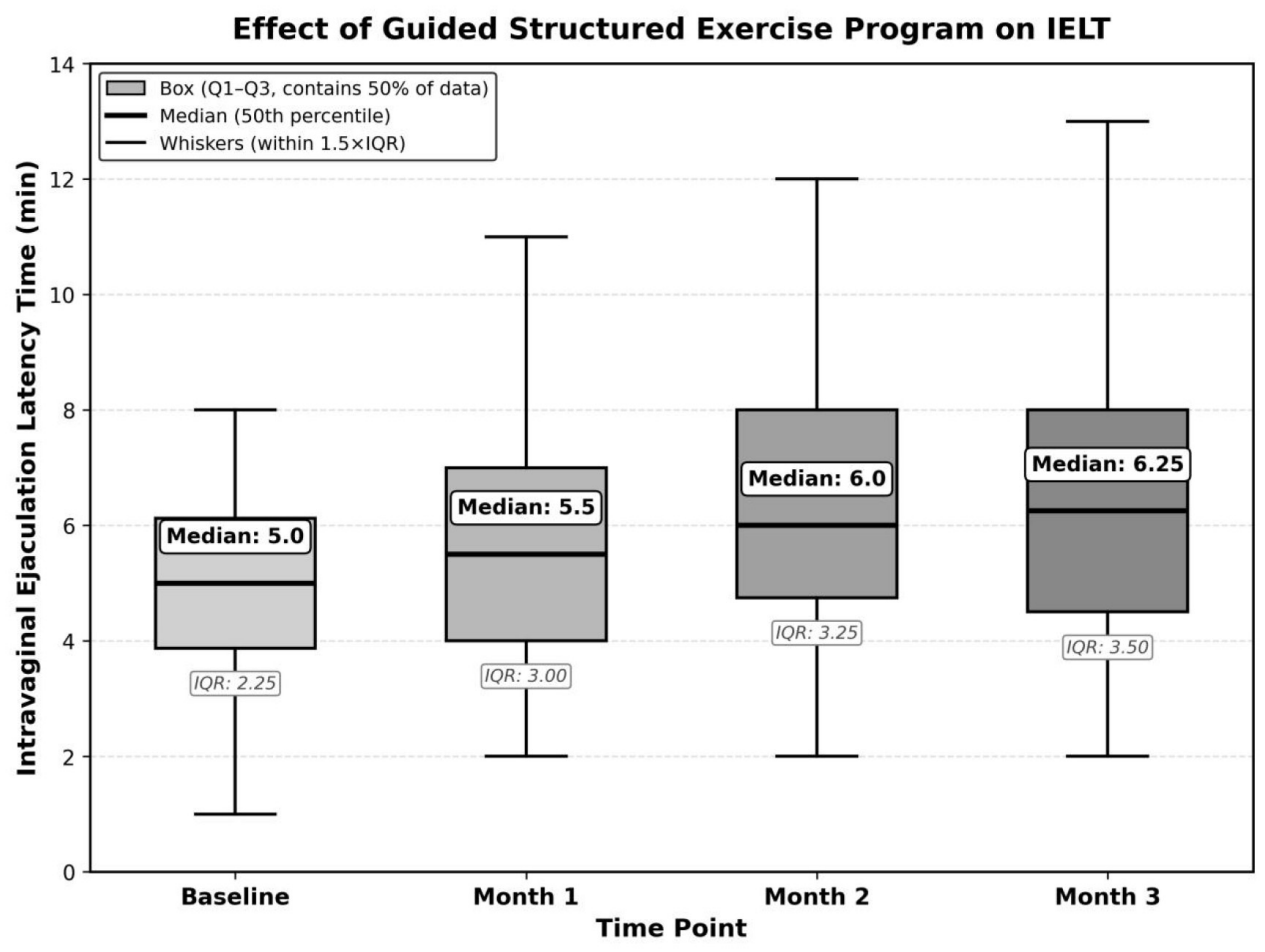
Changes in IELT across the 3-month structured exercise program intervention (baseline, months 1–3). Boxplots show the distribution of self-reported IELT at baseline and at months 1, 2, and 3 among adherent non-athletes (*n* = 20). The box represents the interquartile range (25th−75th percentiles), the central line indicates the median, and whiskers extend to 1.5 × IQR.

The boxplot (Figure 4) illustrates the distribution of IELT at baseline and after 1, 2, and 3 months of the exercise intervention. The box represents the interquartile range (IQR; 25th−75th percentiles), the horizontal line within the box indicates the median, and the whiskers extend to 1.5 × IQR. At baseline, the median IELT was 5.0 min (IQR: 3.75–6.0; range: 1–12 min); after 1 month, 5.5 min (IQR: 4.0–7.0; range: 2–11 min); after 2 months, 6.0 min (IQR: 4.75–8.0; range: 2–12 min); and after 3 months, 6.25 min (IQR: 4.75–8.25; range: 2–13 min).

These findings indicate that guided structured exercise program may significantly improve ejaculatory control among participants; however, interindividual variability suggests that identifying predictors of response is necessary for personalized treatment. Multivariate linear regression analysis (Table 2) identified exercise frequency as the strongest predictor of IELT.

**Table 2:** Multivariate linear regression model examining predictors of IELT.

Variable	β	SE	95% CI	*P*-value
Exercise frequency	0.41	0.12	0.17–0.65	0.002^*^
Stress score	−0.28	0.11	−0.51–0.06	0.013^*^
Sleep habit	−0.19	0.10	−0.40–0.02	0.078
BMI	0.05	0.08	−0.11–0.21	0.531

^*^P < 0.05.

Standardized β coefficients are shown. Positive β values indicate association with prolonged IELT. Model fit: *R*^2^ = 0.36, Adjusted *R*^2^ = 0.31, *F* (4, 62) = 8.72, *P* < 0.001.

SE, standard error; CI, confidence interval; BMI, body mass index.

While the median IELT was significantly longer in athletes, the prevalence of PE (IELT ≤ 3 min) did not differ significantly between groups (10.0% vs. 24.3%, *P* = 0.201). This suggests that regular exercise is associated with longer ejaculatory latency, but a larger sample is needed to confirm its association with clinically defined PE.

## 4 Discussion

### 4.1 Interpretation within context of limitations

The findings of this study should be interpreted considering its exploratory nature and methodological constraints. A major limitation is the lack of a concurrent non-exercising control group during the intervention phase; thus, the observed IELT improvements could partially be attributed to placebo effects, natural variation, or repeated measurement effects. The modest sample size and lack of a randomized control group in the intervention phase preclude definitive causal conclusions. The observed improvement in IELT among adherent non-athletes, while statistically significant, requires confirmation in larger, controlled trials. Furthermore, the self-reported nature of both the primary outcome (IELT) and the exercise adherence data introduces potential bias. Despite these limitations, the consistency of our findings—linking both habitual athleticism and a targeted structured exercise program intervention to longer IELT—provides a coherent preliminary signal worthy of further investigation.

It is critical to interpret the present findings within the context of the study's exploratory nature and feasibility objectives. Given the absence of a priori sample size calculation (which would require approximately 300 participants per group to detect a clinically meaningful difference) and the substantial attrition rate (46%), this study was not designed or powered to provide definitive answers. Instead, it serves as a hypothesis-generating pilot investigation, offering preliminary effect size estimates and highlighting the practical challenges of implementing unsupervised home-based exercise interventions in this population. These data can inform the design of future definitive trials.

This preliminary study provides initial evidence that the IELT of athletes was significantly longer than that of non-athletes, suggesting that regular physical exercise may play a beneficial role in maintaining male sexual function. This difference is likely attributable to athletes' long-term engagement in moderate-to-vigorous exercise, which improves cardiovascular function, enhances penile blood flow, and promotes neuromuscular coordination, thereby prolonging IELT (13, 14). Additionally, regular exercise can regulate the hypothalamic–pituitary–gonadal axis, maintain optimal testosterone levels, and increase the sexual response threshold, all of which contribute to better ejaculatory control (15, 16). Conversely, non-athletes, who often exhibit insufficient physical activity, irregular sleep patterns, and disrupted daily routines, may experience hormonal fluctuations, metabolic decline, and increased psychological stress, which collectively shorten IELT (17). The present study extended these observations by implementing a specific, structured exercise modality—guided structured exercise program—to directly test its effect on IELT.

In this study, non-athletes who adhered to the 3-month guided structured exercise program demonstrated a significant prolongation of IELT. Several mechanisms may explain this association. First, regular moderate-to-vigorous aerobic exercise can improve cardiorespiratory fitness and endothelial function, which may enhance penile blood flow and sexual response. Second, exercise may modulate neuroendocrine pathways (e.g., the hypothalamic–pituitary–gonadal axis) and reduce stress-related hormonal activation, potentially increasing the threshold for ejaculatory reflex activation. Third, improvements in sleep quality, mood, and perceived self-efficacy associated with regular training may reduce performance anxiety and enhance perceived control during sexual activity. Together, these physiological and psychological pathways provide a plausible explanation for the observed IELT improvement following the exercise intervention.

Psychological factors also play a crucial role in the regulation of IELT. This study found that athletes generally exhibited better mental health, characterized by lower levels of anxiety and tension, which aligns with the well-documented psychological benefits of regular exercise. Previous research has indicated that anxiety, depression, performance-related stress, and relationship tension are all associated with impaired ejaculatory control (18, 19). Regular physical activity reduces cortisol levels, improves sleep quality, and enhances self-efficacy, thereby alleviating psychological stress and promoting better control over ejaculation (20). Therefore, the prolongation of IELT observed in physically active men may result from a dual “body–mind” mechanism: physiological improvements in blood flow and neural regulation on one hand, and psychological enhancements in emotional stability and self-regulation on the other.

Notably, the IELT improvement observed here was associated with a low-intensity, scalable intervention. The intervention implemented in this study was a guided, self-administered home exercise program, rather than intensive clinic-based physiotherapy. The observed improvement in IELT, despite this less intensive format, suggests that simple, education-based structured exercise program are feasible and potentially effective in a young, non-clinical population. This has important implications for public health and sexual wellness promotion, as such programs are low-cost, scalable, and avoid the stigma or access barriers associated with clinical treatment. Beyond exercise, other non-pharmacological strategies have also shown promise in improving ejaculatory control. For instance, a recent systematic review on traditional Chinese medicine (TCM) for PE concluded that Chinese herbal medicine may increase IELT, supporting the value of exploring diverse treatment modalities outside conventional pharmacotherapy (21).

## 5 Conclusion

This study indicates that regular physical activity is associated with longer self-reported IELT. In this exploratory, non-randomized intervention, adherence to the 3-month guided structured exercise program was associated with a statistically significant improvement in self-reported IELT from baseline among previously sedentary university students (median increase from 5.0 to 6.25 min; *P* < 0.001). However, due to the lack of a concurrent control group and the limited sample size with a high attrition rate (46%), these preliminary findings should be interpreted cautiously and require confirmation in larger, adequately powered randomized controlled trials.

These findings highlight guided structured exercise program as a feasible, non-pharmacological candidate strategy that could be integrated into campus health promotion to support male sexual wellbeing. Future research should employ more rigorous designs, including active control groups and objective outcome measures, to confirm efficacy and elucidate underlying mechanisms.

## 6 Study limitations and future directions

### 6.1 Limitations

Despite the promising findings, this study has several limitations that should be considered when interpreting the results.

#### 6.1.1 Sample size, power, and attrition

This was a preliminary, exploratory study with a modest sample size, and no formal a priori sample size calculation was performed. Consequently, the study may be underpowered to detect smaller but clinically meaningful effects, particularly in subgroup analyses such as PE prevalence. As noted by reviewers, a sample size of approximately 300 per group would be required to reliably detect a 10% difference in PE prevalence, which further underscores the preliminary nature of our subgroup analyses. The relatively high attrition rate (~46%) in the intervention group further limits the statistical power and generalizability of the per-protocol findings. Attrition likely reflects the practical challenges of sustaining a self-administered, home-based exercise regimen over 3 months without direct supervision.

#### 6.1.2 Self-reported outcomes

The primary outcome (IELT) and exercise adherence were based on participant self-report, which is susceptible to recall bias and social desirability bias. The use of a single, retrospectively reported average IELT, though common, is less precise than prospective, partner-timed measurements.

#### 6.1.3 Non-randomized comparative design

The cross-sectional comparison between athletes and non-athletes is observational. Although groups were comparable in key demographics, unmeasured confounding factors (e.g., genetic predisposition to athleticism, baseline differences in sexual confidence or relationship dynamics) could account for the observed differences in IELT. Causality in this comparison cannot be inferred.

#### 6.1.4 Specificity of the intervention mechanism

The structured exercise program combined multiple elements (e.g., aerobic and resistance activities) and allowed flexibility in exercise type. While this improves feasibility, it limits our ability to isolate which components (e.g., aerobic volume vs. intensity, or resistance training) contributed most to IELT improvement.

#### 6.1.5 Short-term follow-up and lack of control group (intervention phase)

The intervention duration was 3 months, and the study lacked a non-exercising control group for the intervention phase. Therefore, the long-term sustainability of IELT improvements and the possibility that observed changes were due to placebo effects or natural variation over time cannot be ruled out.

#### 6.1.6 Subjective physical activity assessment

Physical activity level, a key predictor in our analysis, was assessed based on self-reported exercise frequency. While practical, this measure is susceptible to recall and social desirability bias. Future studies would benefit from employing more objective quantification methods, such as accelerometry or detailed metabolic equivalent (MET) hour calculations.

### 6.2 Future research directions and implications

These limitations highlight clear pathways for future research while underscoring the practical implications of our work.

#### 6.2.1 Methodological rigor

Future definitive trials should employ larger, randomized controlled designs with an active or wait-list control group. Outcomes should be measured using prospective, partner-timed stopwatch IELT and objective adherence monitoring (e.g., via digital platforms or brief video verification) to minimize bias.

#### 6.2.2 Mechanistic exploration

Future studies should integrate objective physical activity and fitness assessments (e.g., accelerometry, MET-hours, heart-rate–based training load, or estimated VO_2_max), alongside physiological measures (e.g., hormonal panels) and validated psychological questionnaires. This would help disentangle the biopsychosocial pathways through which regular exercise may improve ejaculatory control.

#### 6.2.3 Optimization of the intervention

Research is needed to determine the optimal exercise “dose” (frequency, intensity, time, and type) for improving ejaculatory control, and to develop personalized prescriptions based on baseline fitness, psychological profile, and adherence patterns.

#### 6.2.4 Clinical and public health translation

Universities and public health initiatives could consider integrating structured exercise and lifestyle counseling into wellness programs to support male sexual health. Clinicians may also encourage regular moderate-to-vigorous physical activity as a low-cost behavioral strategy for young men with concerns about ejaculatory control, alongside other evidence-based treatments when appropriate.

## Data Availability

The original contributions presented in the study are included in the article/supplementary material, further inquiries can be directed to the corresponding author.
